# Evidence of brain metabolism redistribution from neocortex to primitive brain structures in early acute COVID-19 respiratory syndrome

**DOI:** 10.1186/s13550-024-01089-3

**Published:** 2024-03-12

**Authors:** Stephan P. M. Souza, Nicoli Colet, Mariana Fujiwara, Alins P. Fernandes, Natalia Tobar, Sergio S. J. Dertkigil, Maria Emilia S. Takahashi, Bárbara J. Amorim, Lucas S. Silva, Clarissa L. Yasuda, Fernando Cendes, Thiago F. de Souza, Juliano T. Rodrigues, Denise E. Zantut-Wittmann, Celso Dario Ramos

**Affiliations:** 1 Nuclear Medicine Division, Department of Radiology, Faculty of Medical Sciences, University of Campinas, Campinas, São Paulo Brazil; 2 Endocrinology Division, Department of Internal Medicine, Faculty of Medical Sciences, University of Campinas, Campinas, São Paulo Brazil; 3 Department of Radiology, Faculty of Medical Sciences, University of Campinas, Campinas, São Paulo Brazil; 4 Gleb Wataghin Institute of Physics, University of Campinas, Campinas, Brazil; 5 Department of Neurology, Faculty of Medical Sciences, University of Campinas, Campinas, São Paulo Brazil

**Keywords:** Positron emission tomography/computed tomography, FDG, COVID-19, Brain metabolism, Quantification, Metabolism redistribution

## Abstract

**Background:**

Neuropsychiatric sequelae of COVID-19 have been widely documented in patients with severe neurological symptoms during the chronic or subacute phase of the disease. However, it remains unclear whether subclinical changes in brain metabolism can occur early in the acute phase of the disease. The aim of this study was to identify and quantify changes in brain metabolism in patients hospitalized for acute respiratory syndrome due to COVID-19 with no or mild neurological symptoms.

**Results:**

Twenty-three non-intubated patients (13 women; mean age 55.5 ± 12.1 years) hospitalized with positive nasopharyngeal swab test (RT-PCR) for COVID-19, requiring supplemental oxygen and no or mild neurological symptoms were studied. Serum C-reactive protein measured at admission ranged from 6.43 to 189.0 mg/L (mean: 96.9 ± 54.2 mg/L). The mean supplemental oxygen demand was 2.9 ± 1.4 L/min. [^18^F]FDG PET/CT images were acquired with a median of 12 (4–20) days of symptoms. After visual interpretation of the images, semiquantitative analysis of [^18^F]FDG uptake in multiple brain regions was evaluated using dedicated software and the standard deviation (SD) of brain uptake in each region was automatically calculated in comparison with reference values of a normal database. Evolutionarily ancient structures showed positive SD mean values of [^18^F]FDG uptake. Lenticular nuclei were bilaterally hypermetabolic (> 2 SD) in 21/23 (91.3%) patients, and thalamus in 16/23 (69.6%), bilaterally in 11/23 (47.8%). About half of patients showed hypermetabolism in brainstems, 40% in hippocampi, and 30% in cerebellums. In contrast, neocortical regions (frontal, parietal, temporal and occipital lobes) presented negative SD mean values of [^18^F]FDG uptake and hypometabolism (< 2 SD) was observed in up to a third of patients. Associations were found between hypoxia, inflammation, coagulation markers, and [^18^F]FDG uptake in various brain structures.

**Conclusions:**

Brain metabolism is clearly affected during the acute phase of COVID-19 respiratory syndrome in neurologically asymptomatic or oligosymptomatic patients. The most frequent finding is marked hypermetabolism in evolutionary ancient structures such as lenticular nucleus and thalami. Neocortical metabolism was reduced in up to one third of patients, suggesting a redistribution of brain metabolism from the neocortex to evolutionary ancient brain structures in these patients.

## Background

Severe acute respiratory syndrome coronavirus two (SARS-CoV‐2) in coronavirus disease 2019 (COVID‐19) was declared as a pandemic in March 2020 [1]. The typical symptoms of the disease were early described as dry cough with mild fever followed by dyspnea, fatigue, and hypoxemia. The main presenting feature among patients admitted to hospital with COVID-19 was hypoxemic respiratory failure, often requiring respiratory support [2]. As the pandemic continued, evidence emerged to suggest that COVID-19 can cause neurological complications. Accordingly, a high incidence of neurological damage related to the disease was widely documented by anatomic and functional methods [3–9].

Positron emission tomography/computed tomography (PET/CT) with [^18^F]fluordeoxyglucose ([^18^F]FDG) is a highly sensitive method for detecting early functional changes in the brain [10]. Studies using [^18^F]FDG PET/CT have shown that long-term brain effects of post-COVID-19 often exhibit hypometabolic patterns, with about half of the brain scans of patients with neurological symptoms being mildly to severely affected [5]. However, almost all those brain imaging studies have been obtained from patients with severe neurological symptoms during the chronic or subacute phase of the disease [3–9]. Therefore, it is unclear whether subclinical changes in brain metabolism can occur early, during the acute phase of the hypoxemic symptoms of the disease, even in patients who do not present signs or symptoms of neurological involvement or those with only mild symptoms.

The aim of this study was to identify and quantify changes in brain metabolism in patients hospitalized for acute respiratory syndrome due to COVID-19 without severe neurological symptoms.

## Methods

### Patients

This single-center open-label observational study was approved by the local Research Ethics Committee under the number CAAE 33477020.8.0000.5404. All participants were informed about the research and signed the informed consent form.

The study was conducted from July 2020 to November 2020, with patients admitted at the Clinical Hospital of the University of Campinas (UNICAMP), Brazil, with positive nasopharyngeal swab test (reverse transcription polymerase chain reaction: RT-PCR) for COVID-19 and respiratory syndrome, non-intubated, without signs or severe symptoms of neurological involvement.

Only patients who were conscious, cognitively, and mentally normal were included. Patients under 18 years of age or with neurological conditions or symptoms such as severe headache, confusion/delirium, toxic/metabolic encephalopathy, seizure, stroke, hypoxic/ischemic injury, suspected meningitis/encephalitis, or myelopathy/myelitis were not included in the study. Patients with mild headache, anosmia/hyposmia, or ageusia/dysgeusia without other neurological symptoms were included in the study. Only initial patient admissions were included: readmissions were excluded.

### Clinical and neurological examination

Clinical examination including neurological assessments were conducted by general clinicians as part of the Hospital routine for COVID-19 patients. This included medical history and clinical evaluation, comprising assessment of the patient’s cranial nerves, motor and sensory systems, reflexes, and coordination. Evaluation of multiple biochemical parameters was also performed, as listed in Table 1. Due to logistical limitations related to the most acute phase of the pandemic, neuropsychological battery was not performed in these patients. A retrospective evaluation of patient data from medical records was conducted to characterize the long COVID syndrome.

**Table 1:** Demographic and clinical characteristics of the patients

Characteristic		All patients (*n* = 23)
Sex		
	Male; n (%)	13 (56.5%)
	Female; n (%)	10 (43.5%)
Age [years]; mean ± SD; median (IQR)		55.5 ± 12.1; 55 (33–78)
BMI [kg/m^2^]; mean ± SD; median (IQR)		32.5 ± 7.0; 30.7 (22.8–47.8)
Obesity [BMI > 30 kg/m^2^]; n (%)		13 (56.5%)
Tobacco consumption; n (%)		6 (26.1%)
Alcohol consumption; n (%)		12 (52.2%)
Arterial hypertension; n(%)		10 (43.5%)
Symp-days [days]; mean ± SD; median (IQR)		12.9 ± 3.8; 12 (4–20)
CRP [mg/L]; mean ± SD; median (IQR)		96.9 ± 54.2; 83.9 (6.43–189.0)
Lactate dehydrogenase [U/L]; mean ± SD; median (IQR)		323.4 ± 135.5; 294 (137–768)
HbA1c [%]; n (%); mean ± SD; median (IQR)		20 (86.9%); 6.9 ± 2.0; 6.4 (5.3–12.7)
D-dimer [ng/mL]; mean ± SD; median (IQR)		1508 ± 1706: 778 (255–7331)
INR; mean ± SD; median (IQR)		1.0 ± 0.1; 1.0 (0.8 − 1.2)
ESR [mm/h]; mean ± SD; median (IQR)		62 ± 18; 63 (30–96)
Ferritin [ng/mL]; mean ± SD; median (IQR)		1147.6 ± 895.4; 830 (174–3940)
Fibrinogen [mg/dL]; mean ± SD; median (IQR)		805 ± 1263; 501.8 (363.0-6566.0)
O_2_-flow [L/min]; mean ± SD; median (IQR)		2.9 ± 1.4; 3.0 (0.0–5.0)
Saturation of Peripheral Oxygen (SatO_2_)[%]; mean ± SD; median (IQR)		95.4 ± 2.0; 95 (91–98)
PO_2_ /FiO_2_; mean ± SD; median (IQR)		336.2 ± 60.0; 328 (219–528)
Pulmonary CT involvement [%];		
	<25%; n (%)	2 (8.7%)
	25-50%; n (%)	14 (60.9%)
	50-75%; n (%)	7 (30.4%)
	>75%; n (%)	0 (0%)
Neurological symptoms		
	None	13 (56.5%)
	Mild headache	4 (17.4%)
	Anosmia/hyposmia	5 (21.7%)
	Ageusia/dysgeusia	4 (17.4%)

*Abbreviations*: BMI = body mass index; CRP = C reactive protein; CT = computed tomography; ESR = erythrocyte sedimentation rate; HbA1c = glycated hemoglobin; INR = international normalized ratio; IQR = interquartile range; O_2_-flow = oxygen flow rate; SatO_2_ = saturation of peripheral oxygen; PO_2_/FiO_2_ = ratio of mean arterial oxygen tension (PO_2_) and inspired oxygen/fraction (FIO_2_); SD = standard deviation

### [^18^F]FDG PET/CT image acquisition

[^18^F]FDG PET/CT brain imaging of all patients was obtained up to three weeks after the onset of COVID-19 symptoms. Patients fasted for six hours before imaging. Dedicated brain images in three-dimensional mode were acquired for ten minutes using a Biography mCT40 PET/CT scan (Siemens Medical Solutions Inc., Knoxville, Tennessee, USA) 1.5 h after injection of 4.4 MBq/kg of [^18^F]FDG (Cyclobras Radiopharmaceuticals, São Paulo, Brazil).

CT was acquired with 100 kV tube voltage, 120 mA, 12 mm total beam collimation width, table speed of 12.0 mm per gantry rotation, pitch 1.0, matrix 512 × 512, voxel size of 0.4395 × 0.4395 × 0.60 mm. CT images were reconstructed using H31s convolution kernel. No contrast media was injected in the patients.

PET images were reconstructed using ordered subset expectation maximization (3D-OSEM) algorithm incorporating time-of-flight (TOF) model and point spread function (PSF) correction, with four iterations and 21 subsets, 2 mm Gauss Filter. Model-based (relative scatter scaling) was used for scatter correction. CT-based attenuation correction was also performed on PET images. Corrections for dead-time, decay, and randoms were also performed.

### Image processing and interpretation

Visual analysis was performed by two experienced nuclear medicine specialists and one radiologist. Visual reading was the first step for images evaluation. Semiquantitative analysis was performed using MI-Neurology, a Siemens commercial software that is part of the Syngo.via Neurology package (Siemens CTI Molecular Imaging, Knoxville, TN, USA), previously reported for evaluating of various brain disorders [11, 12]. We utilized the FDG2B and FDG4B databases for elderly and younger subjects, respectively, provided by this commercial software, choosing the whole brain for intensity normalization (scaling). The software places images in the same standardized tridimensional space. The standard deviation (SD) from the mean value was achieved by converting the individual cerebral PET image to a standardized uptake value ratio image and comparing it voxel-by-voxel with an age-matched normal database of healthy subjects by using a two-sample t-test. Hypermetabolic or hypometabolic clusters were quantitatively described in terms of severity (Z-score increasing or decreasing in the hypermetabolic or hypometabolic zones, respectively).

**Fig. 1:**
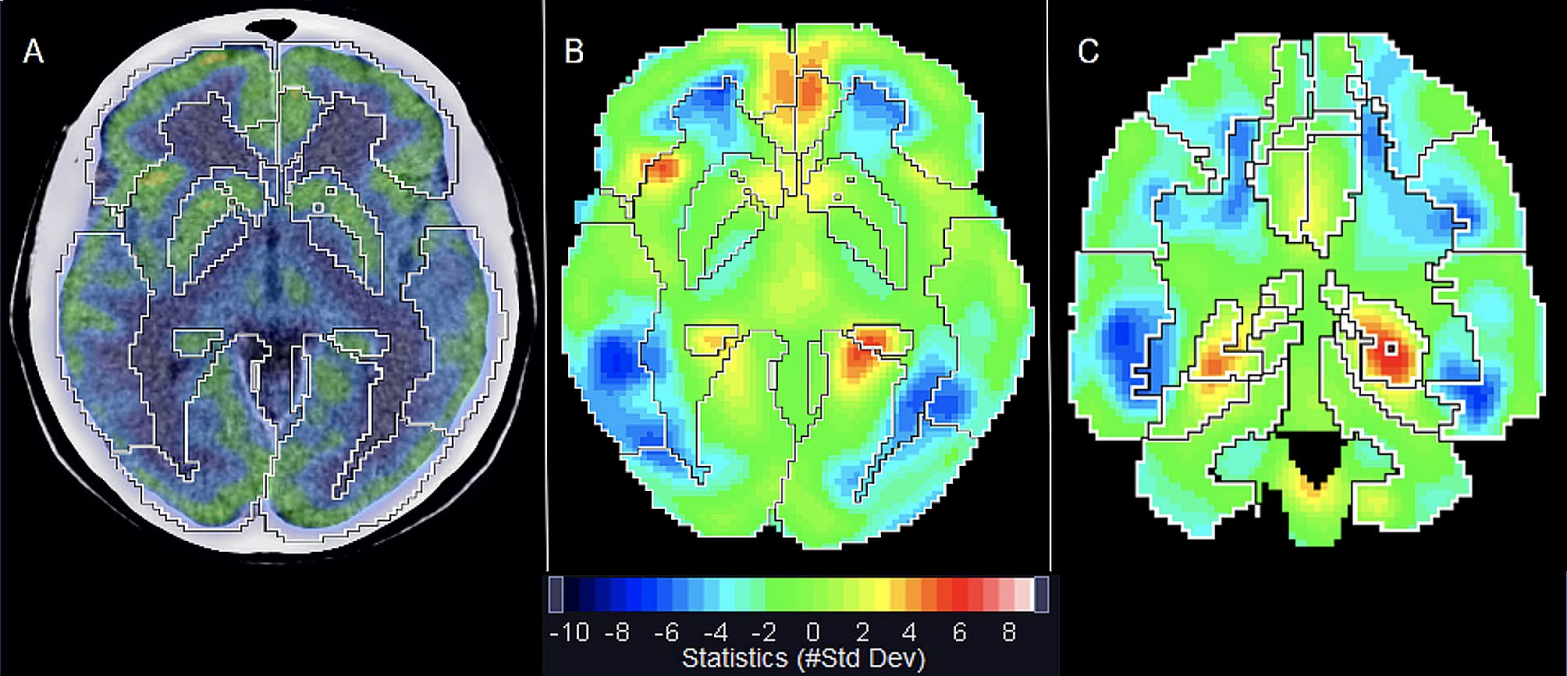
Quantification template for 23 brain regions. The software automatically delineates the various brain regions and performs a voxel-by-voxel statistical comparison of the patient’s [^18^F]FDG PET/CT with a control group. Quantification data is displayed in a solid rainbow color scale as standard deviation (#StdDev) of the mean uptake of a normal database. Regions with increased or reduced glucose metabolism are displayed in red or blue respectively. Fused PET/CT images allow better anatomical recognition of the region of interest (**A**). Images can be read in 3 anatomical planes: axial (**B**), coronal (**C**) and sagittal

The results were depicted in images as well as in a table, expressed as SD units of each patient voxel compared to the mean of the same voxel in the control group. Each semiquantification output was interpreted jointly with the visual reading result, as recommended [13]. [^18^F]FDG uptake was evaluated in 23 brain regions, according to the Combined Automated Anatomical Labeling (C-AAL), including basal ganglia, frontal lobes, parietal lobes, temporal lobes, occipital lobes, caudate nuclei, lenticular nuclei, thalami, hippocampi, cerebellum, brain stem, and olfactory cortices (Fig. 1). More than 2SD above or below the reference values in each area were considered outside normal reference limits. The number of regions outside the reference values was also counted for each patient.

### Statistical analysis

Statistical analysis was performed by the institutional Biostatistics Service, using the SAS System for Windows version 9.4 (SAS Institute Inc, 2002–2008, Cay, NC, USA). To describe the profile of the sample according to the variables under study, categorical variables were presented in absolute frequency values (n) and percentage (%). Descriptive statistics of the numerical variables were also calculated (mean, standard deviation, median, minimum, and maximum values). Data distribution was analyzed using the Shapiro-Wilk normality test. As the normality test rejected the hypothesis of a normal distribution, we utilized Mann-Whitney or Kruskal-Wallis tests for comparisons involving categorical variables. Additionally, the Spearman correlation coefficient was employed to assess the relationship among numerical variables. The significance level adopted for the study was 5% (*p* < 0.05).

## Results

### Patient characteristics

Twenty-three patients (13 male, mean age 55.5 ± 12.1 years) were included in the study. Images were acquired between four and 20 days after the onset of symptoms (mean 12.9 ± 3.8 days). Thirteen patients (56.5%) did not present any neurological symptoms. Ten patients (43.5%) presented only mild headache, anosmia/hyposmia, and/or ageusia/dysgeusia: respectively four, five and four patients.

The mean body mass index (BMI) was 32.5 ± 7.0 kg/m^2^, ranging from 22.8 to 47.8 kg/m^2^, and for 13 patients (56.5%) BMI was > 30 kg/m^2^. Mean C-reactive protein (CRP) measured at admission was 96.9 ± 54.2 mg/L, ranging from 6.43 to 189.0 mg/L. At the time of image acquisition, supplemental oxygen mean demand (O_2_-flow) was 2.9 ± 1.4 L/min and the mean respiratory frequency was 19.7 ± 2.9 breaths/min.

General descriptive analysis of demographic and clinical characteristics of the 23 patients are summarized in Table 1.

### Regional brain metabolism compared to control database

The median SD [min-max] of whole brain glucose metabolism of the 23 patients was within the ± 2SD reference limits (0.8SD [-2.5–3.6]). Frontal, parietal, temporal and occipital lobes presented negative median SD values of [^18^F]FDG uptake, and hippocampus, cerebellum, brain stem, as well as basal ganglia and thalamus showed positive median SD values (Table 2).

**Table 2:** **Regional cerebral [18 F]FDG uptake in COVID-19 patients compared to control subjects**

Brain Area		Uptake intensity (SD of Database SUVmean)	
		mean ± SD	median (IQR)
Whole Brain		0.4 ± 1.4	0.8 (-2.5 to 3.6)
Cerebellum		1.3 ± 1.2	1.0 (-0.6 to 3.9)
Brain Stem		2.1 ± 1.1	1.8 (0.1 to 4.6)
Basal ganglia	Left	3.0 ± 2.0	2.9 (-1.4 to 7.7)
	Right	2.1 ± 1.4	1.8 (-1.1 to 4.5)
Frontal Lobe	Left	-1.1 ± 1.7	-1.1 (-4.2 to 2.3)
	Right	-2.0 ± 2.3	-1.7 (-8.9 to 1.5)
Parietal Lobe	Left	-1.5 ± 1.8	-1.7 (-4.8 to 2.2)
	Right	-1.4 ± 1.4	-1.3 (-3.8 to 0.8)
Temporal Lobe	Left	-1.0 ± 1.2	-1.3 (-2.8 to 1.5)
	Right	-1.0 ± 1.2	-1.2 (-3.3 to 1.6)
Occipital Lobe	Left	-1.0 ± 2.0	-0.7 (-4.6 to 2.4)
	Right	-0.5 ± 1.7	-0.1 (-3.1 to 1.9)
Caudate Nucleus	Left	1.1 ± 1.4	0.9 (-1.5 to 3.9)
	Right	0.6 ± 1.1	0.3 (-1.0 to 3.2)
Lenticular nucleus	Left	4.8 ± 2.9	4.2 (-0.7 to 12.5)
	Right	4.6 ± 2.8	3.5 (0.1 to 11.8)
Thalamus	Left	2.9 ± 2.0	2.9 (-1.5 to 6.2)
	Right	2.1 ± 1.5	1.8 (-1.1 to 5.6)
Hippocampus	Left	1.5 ± 1.7	1.5 (-2.1 to 5.0)
	Right	1.2 ± 1.8	1.3 (-3.7 to 3.3)
Olfactory cortex	Left	0.6 ± 1.6	0.5 (-1.8 to 4.3)
	Right	0.3 ± 1.7	0.4 (-3.0 to 3.6)

*Abbreviations*: SD = standard deviation, SUV = standardized uptake value

**Fig. 2:**
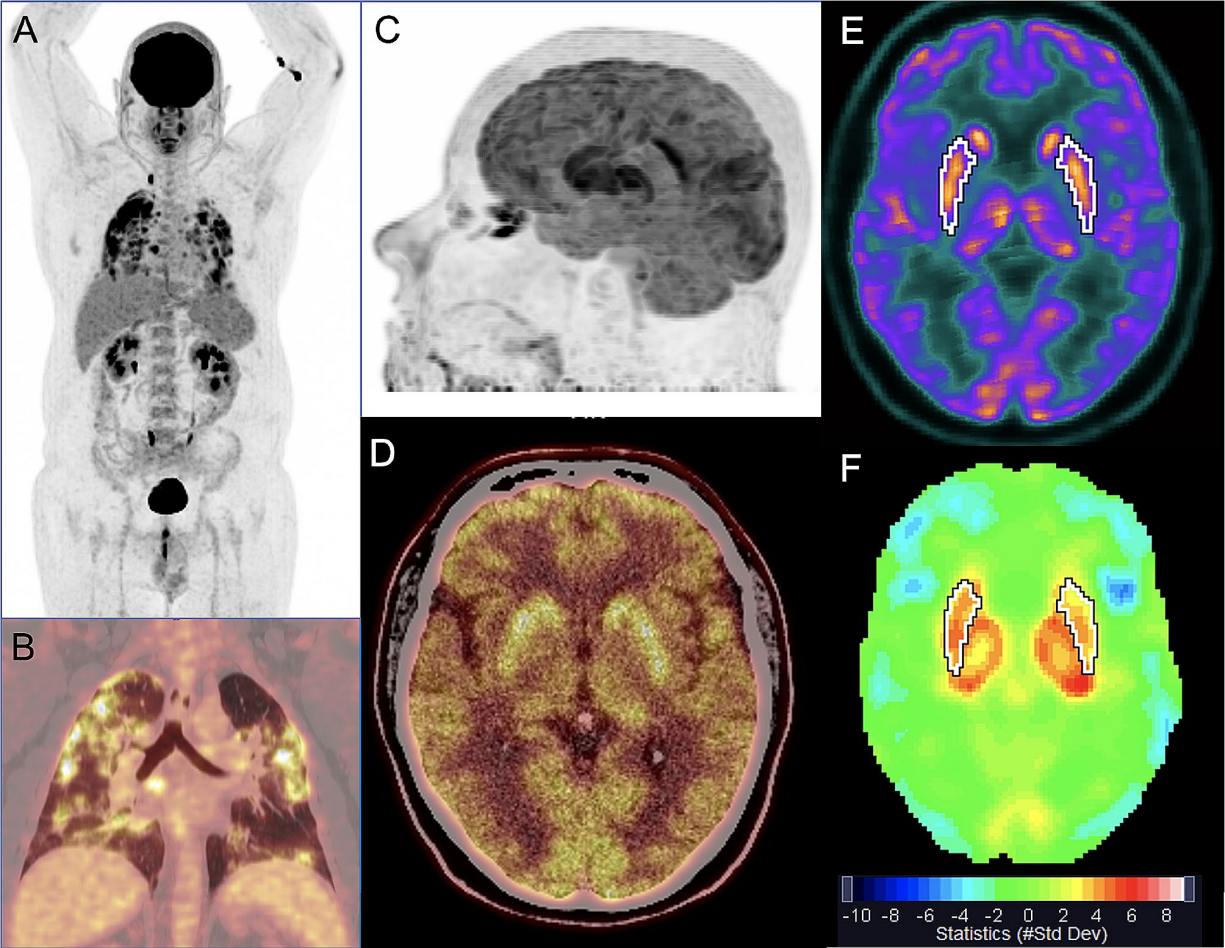
[^18^F]FDG PET/CT of a previously healthy 49-year-old man on the 14th day of acute COVID-19, with respiratory failure and no neurological symptoms, except for anosmia and ageusia. Whole-body maximum intensity projection (MIP) image (**A**) and coronal PET/CT image fusion of the chest (**B**) show marked tracer uptake in the inflamed lungs. MIP (**C**) and axial PET/CT image fusion of the brain (**D**) show marked bilateral tracer uptake in lenticular nuclei. Axial plane of automatic delimitation of lenticular nuclei is shown in **E**. Quantification data displayed on a rainbow color scale as standard deviation (#StdDev) of the mean uptake of a normal database demonstrate increased glucose metabolism in lenticular nuclei and thalami (red in **F**). Quantification values were as high as 5.7 StdDev above the control group on the right lenticular nucleus (4.5 on the left) and 6.2 on the left thalamus (4.4 on the right). Also note mild reduction of brain glucose metabolism in neocortical areas (light blue color in **F**), especially in frontal lobes. The patient progressively recovered, was discharged after 7 days and followed up for 13 months, without presenting any neurological symptom or sequelae

**Fig. 3:**
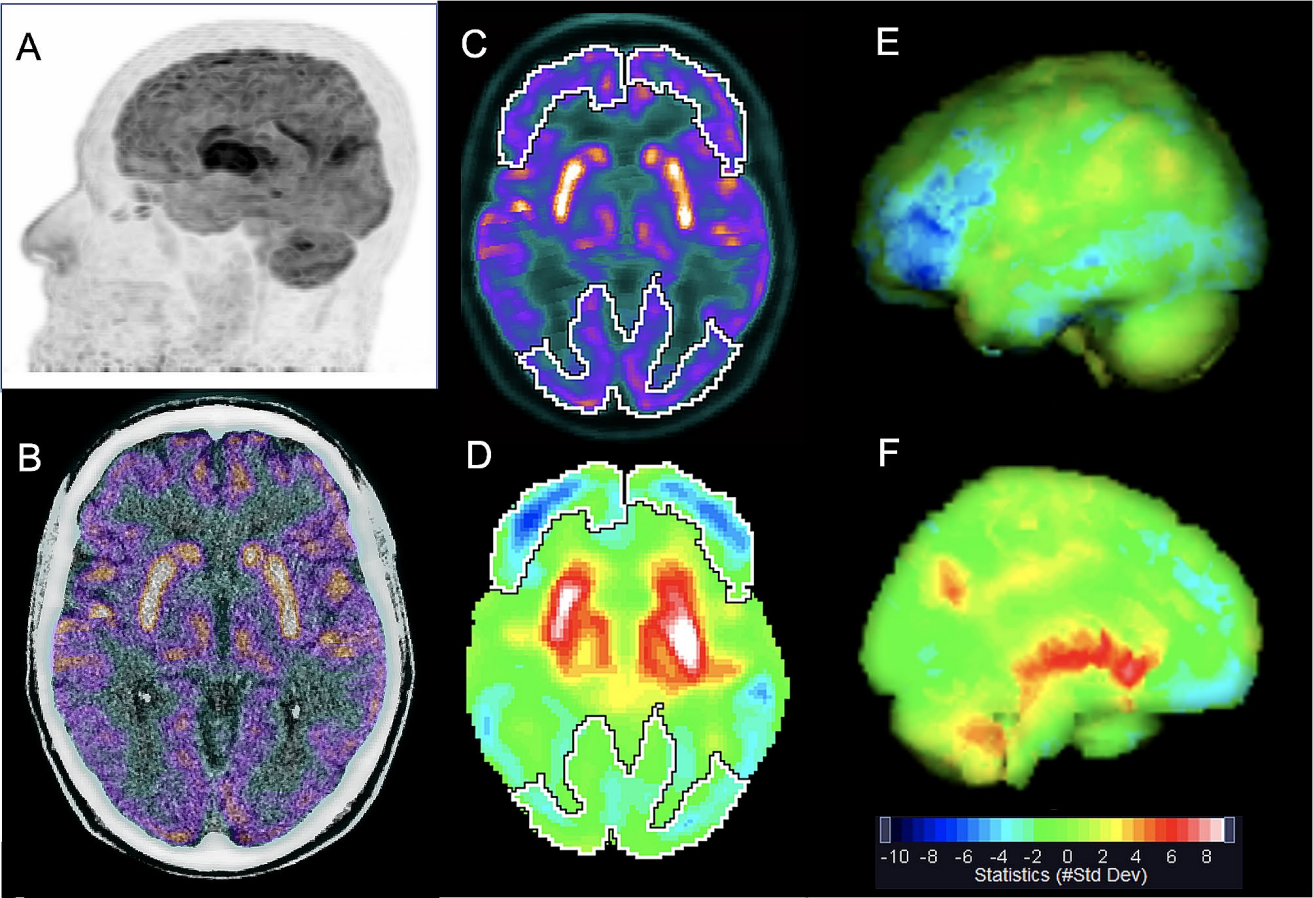
[^18^F]FDG PET/CT of a 69-year-old man on the 20th day of acute COVID-19, with respiratory failure, without neurological symptoms. Maximum intensity projection (MIP) (**A**) and axial PET/CT image fusion of the brain (**B**) show marked bilateral [^18^F]FDG uptake in lenticular nuclei. The axial plane of automatic delimitation of frontal and occipital lobes is shown in **C** and **D**. Quantitative data are displayed in a rainbow color scale as standard deviation (#StdDev) of the mean uptake of a normal database: in an axial plane in **D**, and as three-dimensional surface rendered displays of the left hemisphere of the brain in **E** (lateral view) and **F** (medial view). Reduced metabolism is seen in frontal lobes and temporo-occipital areas (respectively dark blue and light blue) in **A** and **E**. Also note the markedly increased brain metabolism in lenticular nuclei and thalami (red in **D** and **F**), suggesting a redistribution of brain metabolism from the neocortex to evolutionary ancient brain structures. During a 13-month follow up, no neurological symptoms or sequelae were detected. Quantification values (in #StdDev units) are: -4.6 / -2.6, for right / left frontal lobes; -3.3 / -2.4, for right / left temporal lobes; -3.0 / -3.9, for right / left occipital lobes; 11.5 / 12.5, for right / left lenticular nuclei; 3.7 / 4.8, for right / left thalami

The overall median SD [min-max] metabolism of basal ganglia was mildly increased on the left (2.9SD [-1.4 to 7.7]) and close to the upper reference limit on the right (1.8SD [-1.1 to 4.5]). However, when analyzing separately the lenticular and caudate nuclei, a marked increase in median SD metabolism was observed in the former (right: 4.2 SD [-0.7 to 12.5]; left: 3.5SD [0.1 to 11.8]) and normal median SD metabolism in the latter (right: 0.9SD [-1.5 to 3.9]; left: 0.3SD [-1.0 to 3.2]) (Figs. 2 and 3). Median SD metabolism of thalamus was also increased on the left (2.9SD [-1.5 to 6.2]) and close to the upper reference limit on the right (1.8SD [-1.1 to 5.6]). All the other brain regions presented median SD metabolism within normal limits.

### Brain metabolism regions outside the reference values

Twenty/23 (86.9%) presented whole brain glucose metabolism within the ± 2SD reference limits, while two/23 (8.7%) and one/23 (4.3%), respectively, had whole brain metabolism above or below the reference values (Table 3).

**Table 3:** Number of brain regions with glycolytic metabolism outside the reference values

Brain Area		All patients (*n* = 23)		
		High	Low	Normal
Whole Brain		2 (8.7%)	1 (4.3%)	20 (86.9%)
Cerebellum		7 (30.4%)	0 (0%)	16 (69.6%)
Brain Stem		11 (47.8%)	0 (0%)	12 (52.2%)
Basal ganglia	Left	18 (78.3%)	0 (0%)	5 (21.7%)
	Right	11 (47.8%)	0 (0%)	12 (52.2%)
Frontal Lobe	Left	1 (4.3%)	6 (26.1%)	16 (69.6%)
	Right	0 (0%)	8 (34.8%)	15 (65.2%)
Parietal Lobe	Left	1 (4.3%)	8 (34.8%)	14 (60.9%)
	Right	0 (0%)	8 (34.8%)	15 (65.2%)
Temporal Lobe	Left	0 (0%)	5 (21.7%)	18 (78.3%)
	Right	0 (0%)	4 (17.4%)	19 (82.6%)
Occipital Lobe	Left	1 (4.3%)	7 (30.4%)	15 (65.2%)
	Right	0 (0%)	6 (26.1%)	17 (73.9%)
Caudate Nucleus	Left	6 (26.1%)	0 (0%)	17 (73.9%)
	Right	2 (8.7%)	0 (0%)	21 (91.3%)
Lenticular Nucleus	Left	21 (91.3%)	0 (0%)	2 (8.7%)
	Right	21 (91.3%)	0 (0%)	2 (8.7%)
Thalamus	Left	16 (69.6%)	0 (0%)	7 (30.4%)
	Right	11 (47.8%)	0 (0%)	12 (52.2%)
Hippocampus	Left	10 (43.5%)	1 (4.3%)	12 (52.2%)
	Right	9 (39.1%)	1 (4.3%)	13 (56.5%)
Olfactory cortex	Left	5 (21.7%)	0 (0%)	18 (78.3%)
	Right	4 (17.4%)	2 (8.7%)	17 (73.9%)

Whole basal ganglia metabolism was frequently increased (left side in 78.3% of the patients; right side: 47.8%) and normal in the other patients (left side: 21.7%; right: 52.2%). None of the patients showed reduction in basal ganglia metabolism. When basal ganglia were analyzed separately as lenticular and caudate nuclei, a different pattern was found between these two structures. Most patients presented increased metabolism in lenticular nuclei (91.3% of patients in both sides) (Figs. 2 and 3) and also normal metabolism in caudate nuclei (73.9% on the left and 91.3% of patients on the right side) (Table 3).

**Fig. 4:**
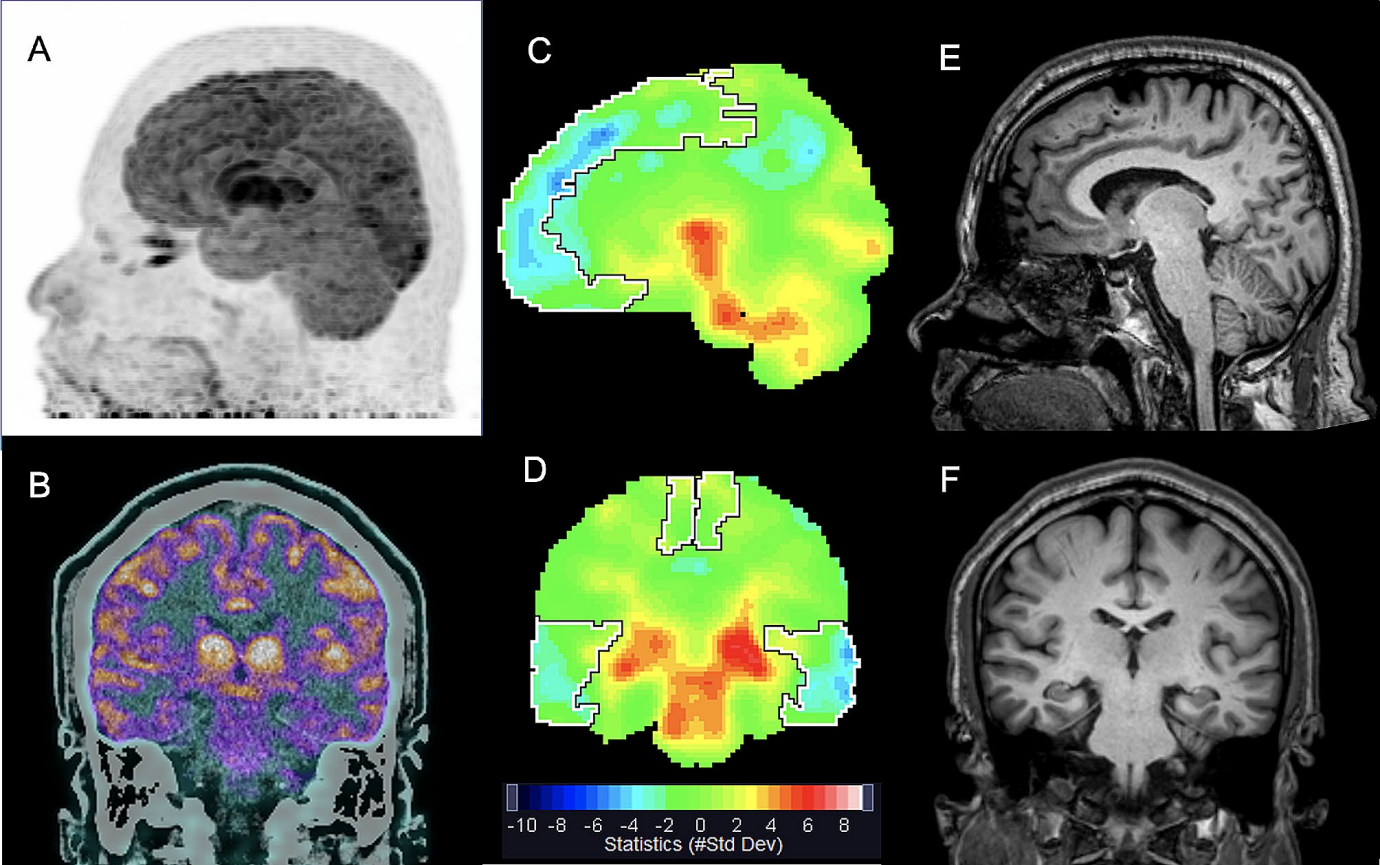
[^18^F]FDG PET/CT of a 43-year-old man on the 12th day of acute COVID-19, with respiratory failure, anosmia and ageusia and no other neurological symptoms. Maximum intensity projection (MIP) (**A**) and coronal PET/CT image fusion of the brain (**B**) show marked bilateral [^18^F]FDG uptake in lenticular nuclei and thalami. Quantitative data are displayed in a rainbow color scale as standard deviation (#StdDev) of the mean uptake of a normal database in **C** (sagittal slice) and **D** (coronal slice), including the automatic delimitation of frontal and temporal lobes. Note the reduced metabolism in the frontal and temporal lobes (blue in **C** and **D**) and increased metabolism in thalami and brainstem (red in **C** and **D**), with an appearance of redistribution of [^18^F]FDG uptake from the neocortex to primitive brain structures. An MRI performed three days after PET/CT was totally normal, as seen in sagittal (**E**) and coronal (**F**) T1-weighted slices. The patient was followed-up for 12 months and no neurological symptoms or sequelae were detected. Quantification results (in #StdDev units): -2.9 / -3.9, for right / left frontal lobes; -2.0 / -2.4, for right / left temporal lobes; 5.6 / 6.0, for right / left thalami; 4.6, for brainstem

Thalamus metabolism was increased in most patients on the left side (69.6%) and in almost half of patients (47.8%) on the right (Figs. 3 and 4).

Overall, PET/CT images showed neocortical metabolism generally preserved: 60.9% to 78.3% of all, frontal, parietal, temporal, and occipital lobes. However, hypometabolism was found in 17.4% to 34.8% of these lobes, more frequently in frontal ones (Table 3; Figs. 2, 3 and 4).

The metabolism of the brain stem was increased in almost half of patients (47.8%) (Fig. 4) and also frequently increased in hippocampus and cerebellum (Table 3). The olfactory cortex was normal in most patients, bilaterally increased in four patients, unilaterally increased in one patient, and unilaterally decreased in only two patients (Table 3).

Of the seven/23 patients with anosmia/hyposmia and/or ageusia/dysgeusia, six had normal metabolism in the olfactory cortex and one presented increased metabolism in this structure. None of them showed reduced metabolism in the olfactory cortex.

### Patient follow-up

One patient died from respiratory complications during his hospital stay. The other 22 patients were discharged in good general condition and without complications, only two of them with mild persistent neurological symptoms at hospital discharge (ageusia and anosmia). Eighteen patients were followed-up for a median of 16 months (one to 32 months). One patient reported persistent anosmia during follow-up, and another reported mild and persistent headache after hospital discharge, suggesting long COVID syndrome. Four/22 patients were unreachable and lost to follow-up.

### Relationship between brain metabolism and neurological symptoms, respiratory and inflammatory parameters

#### Comparative analysis

There was no difference when comparing the median SD of [^18^F]FDG uptake between patients with and without mild neurological symptoms (mild headache, anosmia/hyposmia, or ageusia/dysgeusia) in the evaluated cerebral regions, except for the right lenticular nuclei. In this region, the group of patients without neurological symptoms had a median [min-max] of 4.8SD [3.1SD − 11.8SD], while the group with mild symptoms had a median of 3.2SD [0.1SD − 5.8SD] (Mann-Whitney test; *p* = 0.0287).

The median SD of [^18^F]FDG uptake of the olfactory cortex was different between male and female patients (Mann-Whitney test; *p* = 0.0323).

#### Spearman correlations

A positive correlation was observed between the oxygen flow rate level (O_2_-flow) and both left and right frontal lobes SD of [^18^F]FDG uptake: *r* = 0.5432, *p* = 0.0074) and *r* = 0.5528, *p* = 0.0062, respectively. SD of the right frontal lobe showed an inverse correlation with the interval delay from the initial infection (days after the onset of symptoms) (*r*=-0.5993; *p* = 0.0025). Saturation of peripheral oxygen (SatO_2_) exhibited an inverse correlation with right lenticular nuclei SD (*r*=-0.5309; *p* = 0.0091).

An inverse correlation was observed between leukocyte count and SD of caudate nuclei bilaterally (left: *r*=-0.4413; *p* = 0.0350; and right: *r*=-0.4177; *p* = 0.0473), along with a positive correlation with brainstem (*r* = 0.4746; *p* = 0.0221). CRP level exhibited a positive correlation with the SD of the left olfactory cortex (*r* = 0.4416; *p* = 0.0349). Lactate dehydrogenase (LDH), on the other hand, showed a correlation with SD of right thalamus nuclei (*r*=-0.4784; *p* = 0.0283). Ferritin presented positive correlations with both hippocampi sides (*r* = 0.4946; *p* = 0.0164), brainstem (*r* = 0.4572; *p* = 0.0283), and the whole brain (*r* = 0.5126; *p* = 0.0124).

International normalized ratio (INR) showed correlation with SD of [^18^F]FDG uptake of the following structures: right thalamus nuclei (*r*=-0.4169; *p* = 0.0478), left striatum (*r*=-0.4468; *p* = 0.0325), right striatum (*r*=-0.4857; *p* = 0.0188), left caudate nuclei (*r*=-0.4998; *p* = 0.0152), and right caudate nuclei (*r*=-0.6748; *p* = 4.1278 × 10^− 4^). Prothrombin time exhibited correlations with right thalamus nuclei (*r*=-0.4167; *p* = 0.0479), right striatum (*r*=-0.4439; *p* = 0.0339), as well as left and right caudate nuclei (*r*=-0.4226, *p* = 0.0446 and *r*=-0.5595; *p* = 0.0055). Fibrinogen presented correlations with right thalamus nuclei (*r* = 0.5243; *p* = 0.0102).

**Fig. 5:**
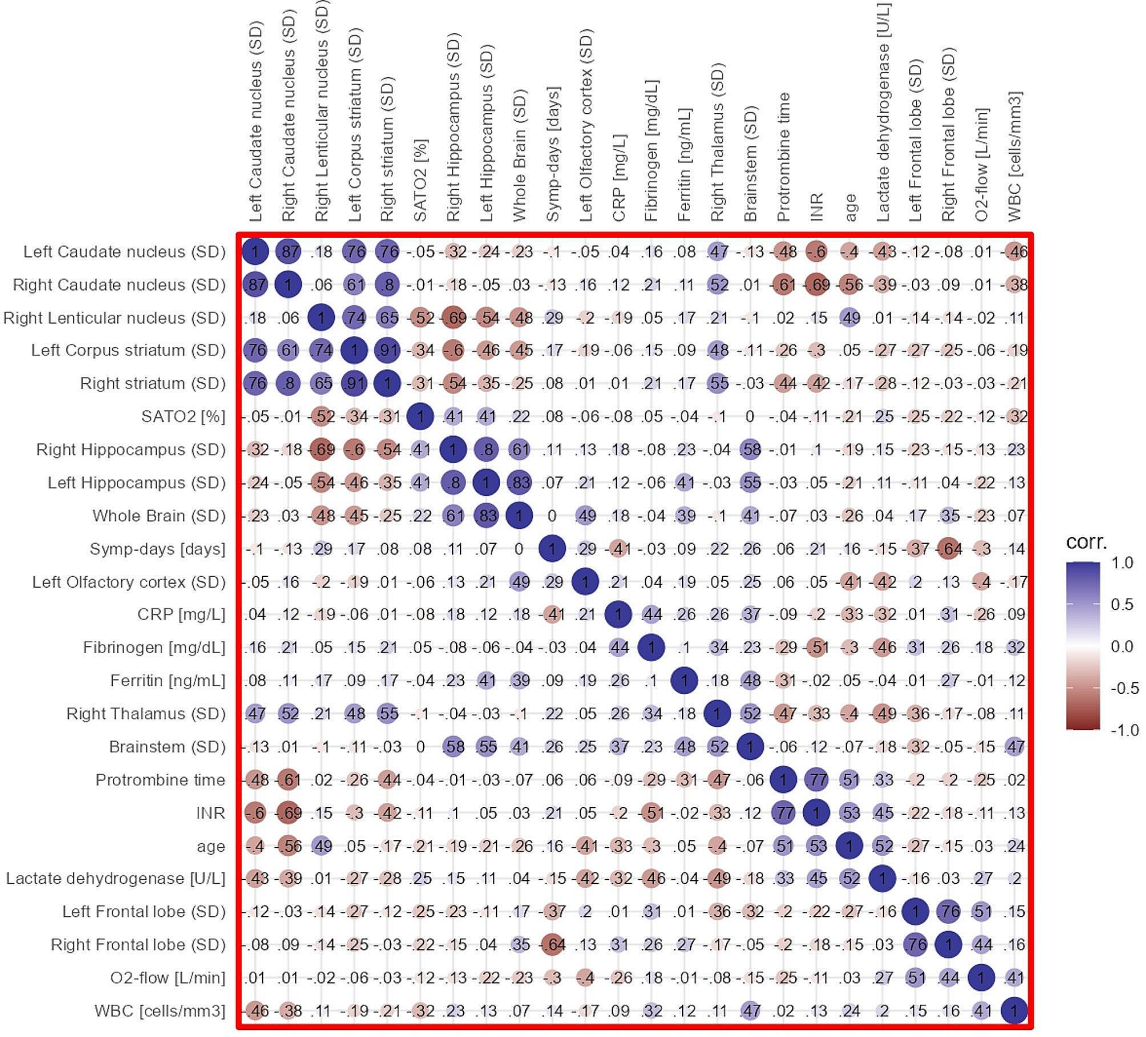
Correlation diagram showing clinical and biochemical data regarding brain findings in acute COVID-19. The color scale indicates the strength of the correlation (blue: correlation + 1.0; red: correlation − 1.0). Abbreviations: SatO2 = saturation of peripheral oxygen; Symp-days = days after the onset of symptoms; CRP = C reactive protein; INR = international normalized ratio; O2-flow = oxygen flow rate; WBC = white blood cells count

There was inverse correlation between patients’ age and the SD of the right thalamus (*r*=-0.5114; *p* = 0.0126) and right caudate nuclei (*r*=-0.4632; *p* = 0.0260). A diagram of the correlations is found in Fig. 5.

## Discussion

Neuropsychiatric sequelae of COVID-19 have been widely documented in patients with severe neurological symptoms during the chronic or subacute phase of the disease, with a plethora of sometimes conflicting imaging results [3, 4, 14].

The present study demonstrates that patients hospitalized for acute COVID-19, requiring oxygen supplementation, may exhibit significant metabolic alterations in specific brain regions. Nevertheless, these alterations do not appear to have a substantial clinical impact in most patients, as none of our patients displayed severe neurological symptoms upon hospital discharge, nor did they experience any relevant sequelae during follow-up. In this sense, only two of them presented symptoms that could suggest long COVID syndrome, preventing the establishment of a relationship with the brain changes found in the initial phase of the disease.

It is worth noting that our group of patients primarily exhibited hypermetabolism in ancient brain structures (such as the lenticular nucleus, thalamus, brainstem, hippocampus, and cerebellum), while experiencing hypometabolism in evolutionarily newer brain regions (frontal, parietal, temporal, and occipital lobes). Notably, all neocortical areas displayed negative median values of [^18^F]FDG uptake, in contrast to all evolutionarily ancient structures showing positive mean values. While it is not possible to definitively establish a causal relationship between these findings, this observation suggests a potential redistribution of brain metabolism during early COVID-19-related hypoxia. This could be seen as a plausible physiological protective mechanism in response to hypoxia or viral/autoimmune cerebral aggression. The oldest parts of the brain are crucial for survival and must be preserved to maintain essential functions for life. While redistribution of perfusion or metabolism within the brain has not been explicitly proposed before, blood redistribution is a recognized physiological protective mechanism across various animal species when considering the body as a whole [15, 16]. Indeed, redistributing cardiac output in response to acute hypoxemia is necessary to ensure perfusion of vital organs, including the brain, heart, and adrenal glands [16]. The brain is an organ with one of the highest oxygen and glucose demands, yet it lacks the ability to store metabolic products for later use [17]. Therefore, during hypoxemia or acute viral/autoimmune COVID-19 insults to the brain, the metabolic redistribution suggested by our findings may contribute to preserving essential brain functions. Of course, further studies are needed to confirm whether this hypothesized redistribution indeed occurs in COVID-19 or even in other infections and brain-related disorders.

The presence of hypermetabolism in certain brain structures has been suggested to be potentially an artifact in COVID-19 patients with neocortical hypometabolism and quantification performed using normalization through whole-brain proportional scaling [3, 4]. Some authors consider that, with the possible exception of concomitant autoimmune encephalitis, it is questionable whether regions of hypermetabolism reported in several studies exhibit true hypermetabolism or are influenced by a quantification artifact related to the normalization process [3, 4]. On the other hand, other researchers do affirm that [^18^F]FDG-PET/CT can be employed to identify hypermetabolism at earlier stages during the acute phase of COVID-19 [18, 19]. Indeed, a visual examination of brain-dedicated and whole-body images of our patients suggested apparently normal global brain [^18^F]FDG uptake. Therefore, as proposed by Guedj et al. [18], discussion of hypermetabolism should focus on its pathophysiological significance, particularly in diseases where brain inflammation can be expected or in disorders still in the clinical and pathological definition phase. In the present study, even if there is an artifactual overestimation of areas with higher uptake, the observed phenomenon of redistributed brain metabolism remains evident.

Specifically, the lenticular nuclei exhibited bilateral hypermetabolism in over 90% of patients with either no or mild neurological symptoms. The median SD of [^18^F]FDG uptake in this structure exceeded expected levels by up to four times when compared to a database of normal subjects, and in one patient, it reached a remarkable 12 SD above normal, bilaterally. Hypermetabolic thalami were also a prevalent finding, identified in 70% of all patients, with bilateral occurrence in 50% of them. Although median values in other brain regions fell within normal limits, hypermetabolism was noted in approximately half of the brainstems, 40% of hippocampi, and 30% of the cerebellums. Neocortical metabolism was generally preserved, but hypometabolism was found in up to one third of all frontal, parietal, temporal and occipital lobes. Few articles discussing PET imaging for acute brain effects of COVID-19 are mostly in agreement with our findings [3, 4, 18, 19].

Different hypotheses could explain these findings, including hypoxic brain injury, direct SARS-CoV-2 brain infection, inflammation and autoimmune-mediated neurological damage.

SARS-CoV-2 predominantly affects the lungs, and the resulting systemic hypoxia is one of the major clinical manifestations of COVID-19 [20]. Brain neurons are particularly vulnerable to reductions in oxygen saturation level and hypoxia has been proposed as a possible contributing cause of neuronal damage in severe SARS-CoV-2 infection [21]. Accordingly, we found associations between O_2_-flow or SatO_2_ and [^18^F]FDG uptake in some brain structures of our patients such as right frontal lobe and right lenticular nucleus. Coagulation markers, such as INR, prothrombin time and fibrinogen were also associated with SD of [^18^F]FDG uptake in thalamus and caudate nuclei.

Different areas of the brain have varying degrees of vulnerability to hypoxic-ischemic insults. The high concentration of mitochondria in basal ganglia neurons predisposes them to ischemic damage [22]. It has been already demonstrated that basal ganglia and thalami may be particularly affected by hypoxia in children and neonates [23, 24]. In both children and adults, hypoxic-ischemic encephalopathy almost invariably causes bilateral and symmetrical damage, often affecting the basal ganglia [25], as observed in our patients with COVID-19.

Similar to our observations, Batista et al. [26] described hypermetabolism in the basal ganglia shortly after hypoxic-ischemic injury in the neonatal period. This was followed by severe hypometabolism in the lenticular nuclei and thalami when the child was restudied several years later [26]. Bilateral lesions of globus pallidus and lentiform/caudate nuclei have also been respectively described in two patients with COVID-19 [27, 28]. Delorme et al. [29] also reported cases of early symptomatic encephalopathy related to COVID-19, with hypermetabolism in the striatum and cerebellar vermis and hypometabolism in neocortical areas, with imaging findings similar to ours.

We also found association between [^18^F]FDG uptake in some structures (caudate nuclei, brainstem, olfactory cortex, thalamus and hippocampi) and inflammatory markers such as leukocyte count, CRP, LDH and ferritin. Direct SARS-CoV-2 brain infection, inflammation and autoimmune-mediated neurological damage could explain these findings. The ACE-2 receptor, the primary target for SARS-CoV-2, is not only expressed in lung epithelia but also in diverse organs and tissues, including the cerebral cortex, striatum, brainstem, choroid plexus, paraventricular nuclei of the thalamus, middle temporal gyrus, and posterior cingulate gyrus [30].

Neuroinvasion via the olfactory tract has also been proposed [31]. Singhal et al. [32] demonstrated evidence of herpes simplex virus dissemination in the brain via the olfactory system and detected focal hypermetabolism in the ventral striatum and septal area using [^18^F]FDG PET. Other FDG PET/CT reports on acute encephalitis suggests that direct viral damage causes focal areas of hypermetabolism presumably due to inflammation, associated with cortical areas of secondary hypometabolism, such as in measles and herpesvirus encephalitis [33, 34]. In COVID-19, the cascade of systemic reactions, like the overproduction of cytokines, as a hallmark of the cytokine storm, may also contribute to the neurotoxic effects [35]. Autoimmune-mediated neurologic damage is recognized in several infections. Encephalitis due to herpes simplex virus 1 is associated with the development of N-methyl-D-aspartate receptor-encephalitis [36]. Basal ganglia hypermetabolism has been consistently reported in Sydenham chorea [37], probably related to antibody-mediated cell signaling [37, 38]. Bilateral striatal hypermetabolism has also been demonstrated in anti-voltage-gated potassium channel complex (VGKCC) antibodies limbic encephalitis [39] and in the immune-mediated IgG4-related disease [40]. Putaminal and cerebellar hypermetabolism was reported in a patient with concomitant autoimmune encephalitis and severe COVID-19 [41].

This study has several limitations, most of them related to restrictions caused by the particularly harsh period of the pandemics in which it was carried out. Although it was conducted prospectively, close follow-up of all patients or interval PET/CT scans were not possible. The brain analysis was based on a commercial software using external database for controls, since a local database was not available. Identifying hypermetabolism using proportional scaling might also have been a limiting factor. About half of our patients were obese, which could have interfered with brain [^18^F]FDG uptake and quantification. The investigation of autoantibodies and the study of the cerebrospinal fluid could have brought useful information about the pathophysiological scenario, but this was also not possible for ethical reasons. Nevertheless, it was feasible to obtain significant insights into an aspect that remains poorly comprehended, namely, the impact of early COVID-19 on brain metabolism. As there is currently little neuroimaging data available at the acute stage of the infection, such findings could help to understand post-acute impairment and sequelae.

## Conclusions

During the acute phase of SARS-CoV-2 infection, alterations in brain metabolism are evident in neurologically asymptomatic or oligosymptomatic hospitalized oxygen-dependent patients. The most prominent changes include heightened metabolism in the lenticular nucleus and thalamus, as well as increased activity in the brainstem, hippocampi, and cerebellum. Furthermore, reduction of neocortical metabolism is frequent in these patients, suggesting redistribution of brain metabolic processes from the neocortex to primitive brain structures. This redistribution could serve as a physiological protective mechanism preserving essential brain functions during the early stages of COVID-19-related respiratory failure.

## Data Availability

The data that support the findings of this study are available from the corresponding author on reasonable upon reasonable request. Data are located in controlled access data storage at Faculty of Medical Sciences, University of Campinas.

## Abbreviations

**[**^**18**^**F]FDG**: [fluorine-18]fluordeoxyglucose
**BMI**: body mass index
**C-AAL**: Combined Automated Anatomical Labeling
**COVID-19**: coronavirus disease 2019
**CRP**: C-reactive protein
**O**_**2**_**-flow**: supplemental oxygen mean demand
**OSEM**: ordered subset expectation maximization
**PET/CT**: positron emission tomography/computed tomography
**PSF**: point spread function
**RT-PCR**: reverse transcription polymerase chain reaction
**SARS-CoV‐2**: Severe acute respiratory syndrome coronavirus two
**SD**: standard deviation
**SUV**: standardized uptake value
**TOF**: time-of-flight
**VGKCC**: voltage-gated potassium channel complex
